# Structural features of DNA and their potential contribution to blind mole rat (*Nannospalax xanthodon*) longevity

**DOI:** 10.1007/s10522-025-10221-2

**Published:** 2025-03-25

**Authors:** Gamzenur Sönmez, Tuba Yağcı Gurbanov

**Affiliations:** 1 Department of Molecular Biology, Institute of Graduate Education, Bilecik Şeyh Edebali University, Bilecik, Türkiye; 2 Department of Molecular Biology and Genetics, Faculty of Science, Bilecik Şeyh Edebali University, Bilecik, Türkiye

**Keywords:** Aging, DNA structure, FTIR spectroscopy, PCA, *Nannospalax xanthodon*, *Rattus rattus*

## Abstract

Recent research has shifted the focus from the genetic code of DNA to its structural variations, which significantly impact cancer, genetic diseases, and gene regulation. Structural changes, such as the transition from B-DNA to A-DNA, influence DNA stability and flexibility and are affected by factors like DNA methylation and sugar puckering. This study is the first to investigate the relationship between DNA conformational changes and lifespan in two rodent species. The analysis focused on long-lived *Nannospalax xanthodon* and shorter-lived *Rattus rattus*, utilizing infrared spectroscopy and principal component analysis (PCA) to examine liver DNA. Results indicated that transition from B-form to A- and Z-forms were more prevalent in *N. xanthodon* than in *R. rattus*. However, the dominant DNA conformations in both species are in B-form. Additionally, N-type sugar puckers (C3-endo conformation), associated with these DNA forms, were more prominent in *N. xanthodon*. In contrast, S-type sugar puckers (C2-endo conformation), characteristic of B-DNA, were found at lower levels in *N. xanthodon*. Furthermore, the variations in methylation-specific structural modifications of nucleobases were quantitatively assessed among these species. The study proposes a significant connection between the long lifespan of *N. xanthodon*, which live underground, and their unique DNA structure, offering insights into how different DNA forms, as well as the conformations of their backbone and sugar-base components, may affect longevity, highlighting potential research avenues regarding the biomolecular aspects of aging.

## Introduction

The structural architecture of DNA, extending beyond its sequence-dependent genetic code, has emerged as a critical determinant of genomic stability, cellular function, and organismal longevity (Watson and Crick 2016). B-DNA, which has a right-handed double helix structure with Watson–Crick base pairing, can form non-B DNA structures such as hairpins, triplexes, cruciform, left-handed Z-forms, G-quadruplexes, and A-motifs under specific conditions (Saenger 1984a). While canonical B-form DNA represents the classical double-helical structure, dynamic conformational shifts, such as transitions to A- or Z-DNA alter biochemical properties like flexibility, stability, and protein interactions, with profound implications for aging and disease (Rich et al. 1983; Saenger 1984b; Mirkin 2007; Bacolla and Wells 2009; Vijg and Suh 2013; Bhanjadeo et al. 2022; Nial and Subudhi 2024).

Recent studies highlight how epigenetic modifications, sugar pucker dynamics, and helical polymorphisms modulate the structural landscape of DNA, potentially influencing stress resilience and lifespan (Sen et al. 2016; López-Otín et al. 2023). DNA methylation and methyl-sugar interactions significantly influence DNA structure and flexibility (Bird 1986; Kumar et al. 2018). During the transition from B-DNA to A-DNA, base pairs shift relative to the helical axis. Additionally, the S-type sugar puckering characteristic of B-DNA shifts toward the N-type sugar puckering associated with A-DNA, resulting in a more flexible structure in A-DNA compared to B-DNA (Saenger 1984b; Ghosh and Bansal 2003). Hence, epigenetic modifications and alternative DNA structures have been highlighted as critical factors influencing complex biological processes at the molecular level, providing insights into cancer and aging mechanisms (Pérez et al. 2022; Winnefeld and Lyko 2023).

Epigenetic research has emerged as a burgeoning field of scientific inquiry, with murine and human studies offering novel insights into unresolved mechanisms of aging and oncogenesis (Mangelinck and Mann 2021). Investigations into mammalian DNA methylation have elucidated its role in critical biological processes, including embryonic development, chromatin dynamics, senescence, and carcinogenic pathways (Liu et al. 2003). Notably, certain species, such as bats, exhibit exceptional longevity and negligible phenotypic manifestations of aging. Comparative analyses of DNA methylation profiles have identified age-associated epigenetic modifications, suggesting that methylation dynamics may mechanistically underpin both extended lifespan and cancer resistance in these species (Wilkinson et al. 2021). The remarkable diversity in mammalian lifespans, ranging from *Mus musculus* (2 years) to cetaceans such as bowhead whales (211 years), provides a unique framework for probing genetic and epigenetic regulators of aging and age-related pathologies, including cancer. Some species belonging to the order Rodentia are the most widely used models in comparative studies on aging in mammals (Lorenzini et al. 2009). Among these, *Heterocephalus glaber* is of particular interest due to its pronounced cancer resistance, attenuated senescence, and lifespan exceeding 20 years, defying traditional mammalian allometric predictions (Gorbunova et al. 2014). Similarly, blind mole-rats of the subfamily Spalacinae (genera *Spalax* and *Nannospalax)* with lifespans exceeding 20 years, challenge conventional mammalian aging paradigms by displaying remarkable cancer resistance and hypoxia tolerance (Shams et al. 2005; Manov et al. 2013; Fang et al. 2014b; Gorbunova et al. 2014; Bugarski-Stanojević et al. 2024). The subterranean rodent species *Nannospalax xanthodon* and *Spalax ehrenbergi* are notable model organisms in biomedical research, characterized by hypoxia tolerance, exceptional longevity (exceeding 20 years), and tumor-suppressive mechanisms. These traits position them as critical systems for investigating evolutionary adaptations to extreme environmental stressors and potential mechanistic pathways for cancer resistance (Manov et al. 2013; Solak et al. 2023). In contrast, shorter-lived species like *Rattus rattus* lack such adaptations, offering a compelling comparative model to probe DNA structure-lifespan relationships (Buffenstein 2005; Edrey et al. 2011; Fang et al. 2014a) These species collectively underscore the utility of comparative epigenetics in advancing our understanding of aging and oncological resilience.

Fourier-transform infrared (FTIR) spectroscopy has emerged as a valuable tool in nucleic acid research, offering insights into DNA structure, conformation, and interactions. FTIR spectroscopy allows for the characterization of various DNA forms, including A, B, and Z-DNA, by analyzing vibrational modes sensitive to base pairing, base stacking, and sugar pucker (Banyay et al. 2003; Gurbanov et al. 2019). Specifically, marker bands in the infrared (IR) spectrum can distinguish between different helical forms and provide information on the overall structural integrity of DNA (Muntean et al. 2013). Furthermore, it has been applied to study DNA methylation, a crucial epigenetic modification (Gurbanov et al. 2019). Subtle spectral changes induced by methylation, such as shifts in bands associated with cytosine and guanine vibrations, can be detected and used to investigate the impact of methylation on DNA structure and stability (Banyay and Gräslund 2002). The technique's sensitivity to global and local structural changes makes FTIR spectroscopy a powerful tool for investigating the diverse roles of DNA in biological processes (Whelan et al. 2011, 2014; Karthikeyan et al. 2022; Teker et al. 2024; Tokgoz et al. 2024).

While there is no direct scientific evidence linking DNA backbone and sugar pucker conformations with longevity in *N. xanthodon*, the unique adaptations such as their hypoxia tolerance, cancer resistance, and longevity, suggest that DNA structure and stability may play a role. The structural dynamics of DNA, including backbone flexibility and sugar pucker transitions between C2-endo and C3-endo conformations, influence the interactions of DNA with proteins involved in DNA replication, repair, and transcription (Banyay and Gräslund 2002). These processes are crucial for maintaining genomic stability and integrity, which are known to decline with age and contribute to age-related diseases. Additionally, the compact and stable structure of A-form DNA could contribute to the genomic integrity and stress resistance observed in these rodents. Therefore, it's plausible that variations in DNA backbone and sugar pucker dynamics could affect the efficiency of these processes, potentially influencing an organism's lifespan and resilience to age-related decline. Further research is needed to explore this potential connection and its implications for understanding aging and developing anti-aging therapies.

This study aims to examine the potential correlation between DNA structural characteristics, including backbone and base conformation, and sugar pucker dynamics in long-living *N. xanthodon* spread in Türkiye. The study compares these findings with those from *R. rattus* which have a notably shorter lifespan. The primary objective is to ascertain whether distinct DNA conformations play a role in the longevity observed in *N. xanthodon* using IR spectral analyses combined with principal component analysis (PCA). This study’s findings could help catalyze a paradigm shift in aging research, positioning DNA conformation as a central player in longevity. By decoding the structural secrets of DNA in *N. xanthodon*, we may unlock strategies to not only extend lifespan but also enhance resilience to age-related diseases, ultimately redefining what it means to age healthily.

## Materials and methods

In this study, genomic DNA was extracted from archived liver tissue specimens stored at -80 °C, sourced from six individuals representing the species *N. xanthodon* and *R. rattus*. No animals were euthanized for this research, as all tissues were derived from pre-existing specimens collected under permits granted by the General Directorate of Nature Conservation and National Parks, an agency of the Turkish Ministry of Agriculture and Forestry (Permission No: 72784983-488.04) and Kırıkkale University Local Ethics Committee for Animal Research (Approval No:15/02-15-18). The tissue samples were obtained from three healthy adult male *N. xanthodon* (mean body weight: 240 g) and three adult male *R. rattus* (mean body weight: 130 g) individuals, who were live-trapped in their natural habitats using humane capture protocols. Age estimation was performed using morphometric parameters established in prior field studies, including body mass, tail length, and cranial characteristics, confirming all specimens as reproductively mature adults (Yiǧit et al. 1998; Yağcı and Gurbanov 2019). Before euthanasia, subjects underwent comprehensive health assessments, including evaluation of morphological traits (e.g., absence of cutaneous lesions or injuries) and behavioral indicators of fitness. Post-euthanasia gross anatomical examination of internal organs further verified the absence of pathologies, ensuring the inclusion of only healthy adult individuals in the experimental cohort.

The DNA extraction buffer was formulated with Tris–HCl (10 mM, pH 8), NaCl (0.3 M), SDS (1%), EDTA (10 mM), and urea (4 M). To facilitate protein removal during the DNA isolation process, 10 µl of proteinase K (10 mg/ml) was introduced to each tissue sample. The samples were then incubated in a shaking incubator at 55 °C for two hours at a speed of 160 rpm. To eliminate large fragments, centrifugation was performed sequentially at 10,000 g for 2 min, followed by 10,000 g for 15 min, and again at 10,000 g for 10 min. To remove RNA contaminants, 5 µl of RNase A was added, and the samples were allowed to sit at room temperature for 30 min. Subsequently, an equal volume of acidic phenol (pH 5.27) and chloroform isoamyl alcohol (24:1) was incorporated. For DNA precipitation, an equal volume of isopropanol was added, followed by pipetting. The resulting mixture was washed with an equal volume of 75% ethanol. Finally, to dissolve the DNA pellet, a solution of 10 mM Tris–HCl and 1 mM EDTA was prepared, and 50 µl of this mixture was added to each sample.

The 10 × TBE buffer was prepared using 54 g of TRIS, 20 ml of 0.5 M EDTA (pH 8.0), and 27.5 g of boric acid, followed by the addition of 500 ml of distilled water. To create a 1 × TBE solution, 90 ml of distilled water was mixed with 10 ml of the 10 × TBE buffer. For the preparation of a 0.8% agarose gel, 0.8 g of agarose was combined with 100 ml of TBE, heated in a microwave until fully dissolved, and subsequently, 1 µl of a 10 mg/ml ethidium bromide (EtBr) stock solution was incorporated. A sample of 5 µl of DNA was combined with 1 µl of loading dye and loaded onto the gel. DNA concentrations were quantified using a Nanodrop spectrophotometer.

### FTIR spectral analysis

A 1000 ng of DNA was placed on a Zn/Se crystal of the ATR (Attenuated Total Reflectance) unit (MIRacle, PIKE) and dried for 3 min with inert nitrogen gas (N_2_). The samples were collected in the 4000–650 cm^−1^ spectral range with a resolution of 4 cm^−1^ and 32 scans using an ATR-FTIR spectrometer (Frontier FTIR Spectrometer, Perkin Elmer). The spectra were obtained from three biological replicates, each containing three technical replicates. The acquisition of the spectra was performed using Spectrum One (Perkin Elmer) software, and spectral data analysis was conducted using OPUS 5.5 (Bruker) software (Ardahanlı et al. 2022; Teker et al. 2023; Baba et al. 2024).

In quantitative spectral analyses, the average raw spectra obtained from technical replicates were processed with vector normalization with 9 smoothing points applied to the second derivatives in the fingerprint spectral window (1800–650 cm^−1^) using OPUS 5.5 (Bruker) software. The wavenumbers and absolute intensities of DNA-specific bands in the processed average spectra were analyzed using the peak-picking method. In the standard peak-picking method, analyses were performed with 0% sensitivity, peak minima, and within the spectral window of 1800–650 cm^−1^ (Gurbanov et al. 2018, 2021).

### Principal component analysis

Principal component analysis (PCA) was employed to discriminate between the FTIR spectral profiles of DNA from *N. xanthodon* and *R. rattus*, to identify variables associated with structural variations in DNA. Raw spectral data within the 4000–650 cm⁻^1^ range underwent three preprocessing steps to minimize instrumentation-dependent biases in analyses. First, baseline correction was applied using an offset adjustment method. Second, a Savitzky-Golay second-derivative transformation with a 9-point smoothing window was implemented to enhance spectral resolution. Third, unit vector normalization was performed to standardize amplitude variations. The processed spectra were mean-centered and cross-validated via a six-segment full method, utilizing a calibration set of six samples. PCA, executed through singular value decomposition (SVD) that is the matrix factorization algorithm, was independently applied to two spectral sub-regions: the true fingerprint window (1485–650 cm⁻^1^) and the antisymmetric PO₂⁻ stretching band window (1300–1200 cm⁻^1^). The model was constrained to four principal components to optimize interpretability. Results were visualized through scores plots, highlighting sample clustering, and loadings plots, identifying spectral features driving intergroup discrimination. This approach facilitated the delineation of species-specific DNA structural characteristics while ensuring analytical robustness across instrumental platforms (Yağcı and Gurbanov 2019; Gurbanov et al. 2019; Kumar et al. 2020).

### Statistical analyses

The statistical evaluations and graphical representations of the results were performed using GraphPad Prism 8.01 (GraphPad, USA). The data were analyzed using an unpaired t-test, and significance levels were indicated as P > 0.05 *, P ≤ 0.01 **, P ≤ 0.001 ***, P ≤ 0.0001 ****. The results are expressed as bar diagrams with mean ± standard error of the mean (SEM).

Moreover, parts of whole analysis were used to illuminate the relationships between DNA forms. The relative percentage of each DNA conformation (A-, B-, or Z-form) for a species is calculated as: %_Conformation_ = (I_Conformation_/I_Total_) × 100%. Where: I_Conformation_: Integrated spectral band intensity of specific DNA conformation (A, B, or Z-related band intensities), I_Total_ = I_A_ + I_B_ + I_Z_: Total intensity of all DNA conformations (sum of A, B, and Z band intensities).

ROC analysis was conducted using GraphPad Prism 8.01 to evaluate and validate the discriminatory power of spectral band parameters in distinguishing between two species. A threshold value was established to differentiate the spectral band parameters of the two species effectively. The analysis generated a receiver operating characteristic (ROC) curve, which illustrates the trade-off between sensitivity (true positive rate) and specificity (true negative rate) across various threshold levels, aiding in informed decision-making. Sensitivity refers to the proportion of correctly identified true positives, while specificity represents the proportion of correctly identified true negatives. To assess the overall diagnostic accuracy of the spectral parameters, the mean area under the curve (AUC) and its standard error (SE) were calculated at a 95% confidence interval. AUC values greater than 0.5 indicate discriminatory ability, with higher values suggesting superior performance in differentiating between the species. Only AUC values exceeding 0.5 were considered meaningful in the analysis (Tokgoz et al. 2024).

## Results

**Fig. 1:**
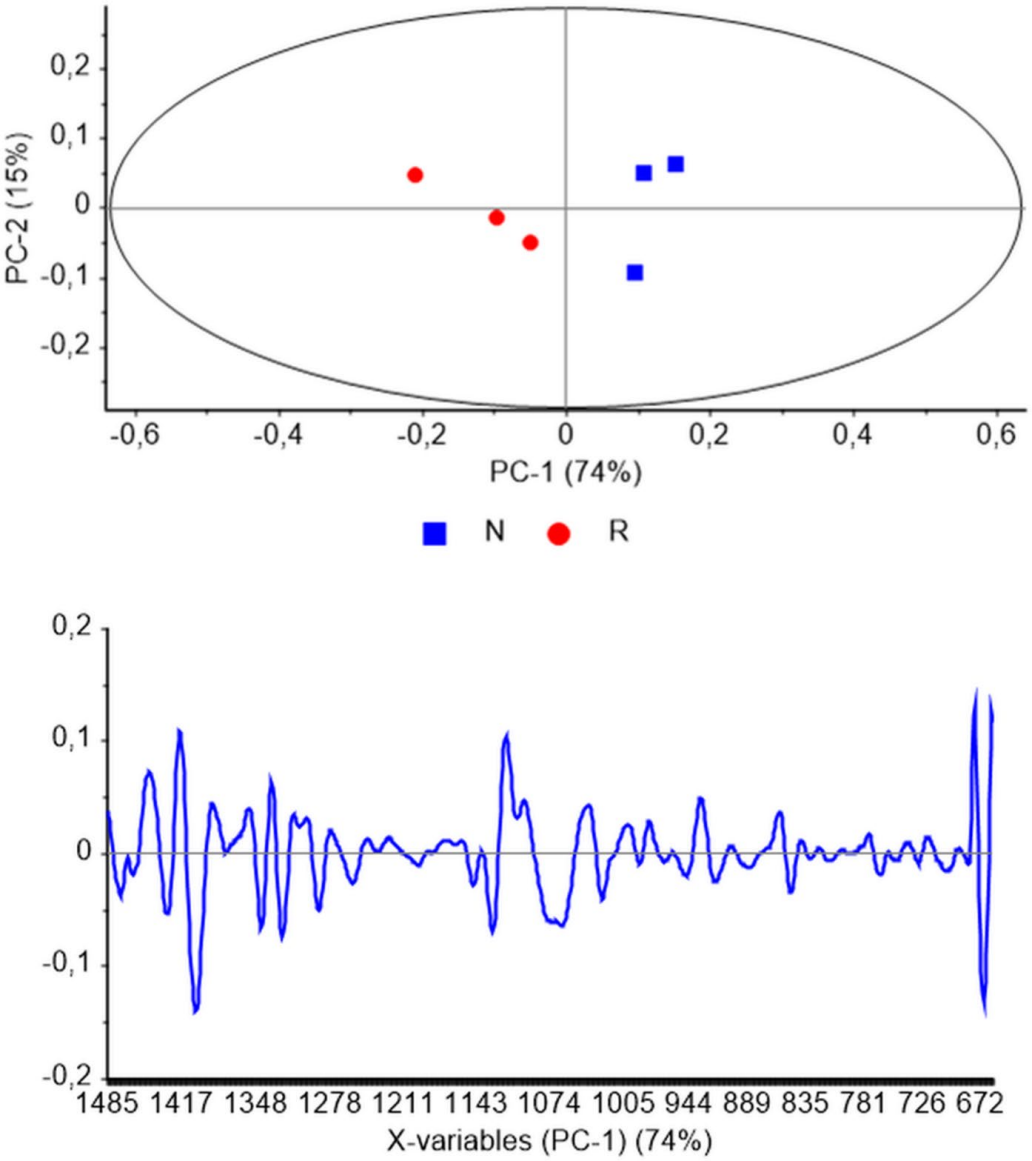
Principal Component Analysis of DNA samples derived from *Nannospalax xanthodon* (N) and *Rattus rattus* (R) liver tissues. The resulting scores and loadings plots were generated within the true fingerprint spectral window (1485–650 cm^−1^)

This study evaluated the structural differences in DNA extracted from liver tissues of two distinct species, *N. xanthodon* and *R. rattus*. To achieve this, an initial exploratory analysis was conducted as a foundational step in understanding the dataset, identifying underlying patterns, detecting anomalies (outliers), and uncovering relationships between variables. Exploratory data analysis is a critical phase in data mining, often serving as the first step in hypothesis generation and providing insights into the structure and characteristics of the data. In this context, PCA, a widely used exploratory data mining tool, was applied to the true fingerprint spectral region (1485–650 cm^−1^) to elucidate major qualitative changes in the DNA structure of the two species (Fig. 1).

The PCA scores plot revealed a clear and complete separation of DNA samples from *N. xanthodon* and *R. rattus* along the PC-1 coordinate, which accounted for 74% of the total variance (Fig. 1, upper panel). Principal components (PCs), particularly PC-1 and PC-2, represent the dominant sources of variation in biological datasets and encapsulate gross molecular variables that contribute to the observed differences (Severcan et al. 2024). The pronounced segregation along PC-1 underscores the significant structural distinctions between the DNA of *N. xanthodon* and *R. rattus*. Complementing the scores plot, the loadings plot (Fig. 1, lower panel) identified specific spectral discriminators responsible for the observed separation. These discriminators correspond to critical molecular parameters, including backbone conformations, sugar puckering modes, and variations in nucleobase composition, all of which are known to influence DNA structure and function.

**Fig. 2:**
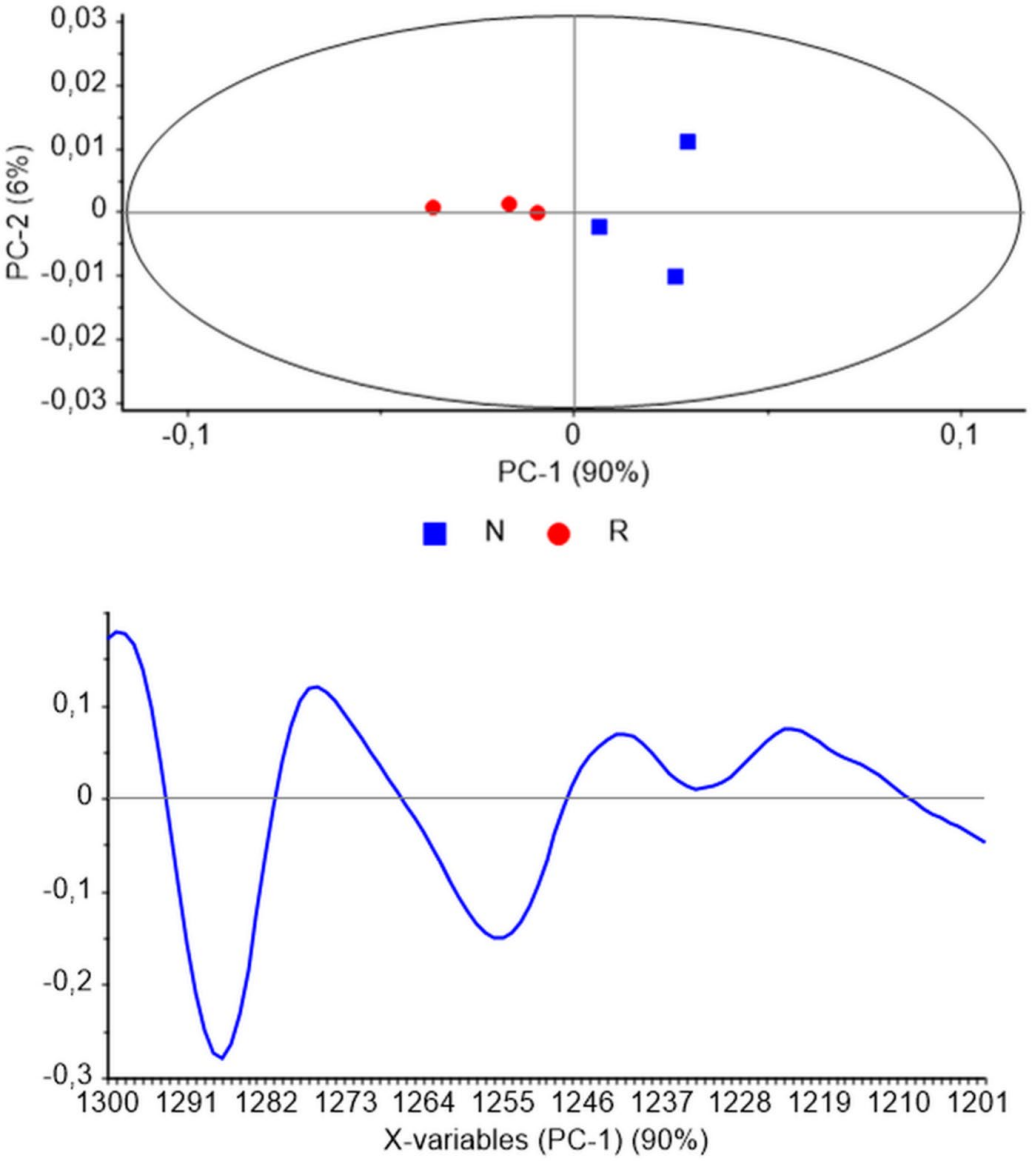
Principal Component Analysis of DNA samples derived from *Nannospalax xanthodon* (N) and *Rattus rattus* (R) liver tissues. The resulting scores and loadings plots were generated within the antisymmetric PO₂⁻ stretching band window (1300–1200 cm⁻^1^)

Further analysis focused on the PO_2_ antisymmetric stretching band region (1300–1200 cm^−1^), which is particularly sensitive to changes in the DNA backbone. PCA applied to this spectral region revealed distinct alterations linked to the structural properties of the DNA backbone (Fig. 2). The scores plot demonstrated a 90% segregation of the samples along the PC-1 axis (Fig. 2, upper panel), indicating that the observed differences were predominantly driven by variations in the backbone structure. The corresponding loadings plot (Fig. 2, lower panel) highlighted key spectral discriminators associated with specific molecular features, including the C5 = C6 ring vibration of cytosine and the conformational differences between the A- and B-forms of DNA. These findings suggest that the structural divergence between the DNA of *N. xanthodon* and *R. rattus* extends beyond mere sequence differences and encompasses significant variations in backbone dynamics and secondary structure.

Collectively, these results underscore the utility of PCA as a powerful tool for discerning subtle yet biologically relevant differences in complex spectral datasets. The observed segregation and identified discriminators provide compelling evidence of pronounced structural disparities in the DNA of the two species, offering valuable insights into their molecular biology and evolutionary divergence..

Following an initial examination of the variables through exploratory analysis, a quantitative peak-picking analysis was subsequently conducted on the dataset. This analytical approach was employed to determine the intensities of specific spectral bands and indices linked to the structural characteristics of DNA. The exploratory phase allowed for a comprehensive understanding of the data's underlying patterns and relationships, ensuring that the subsequent quantitative analysis was both robust and targeted.

**Fig. 3:**
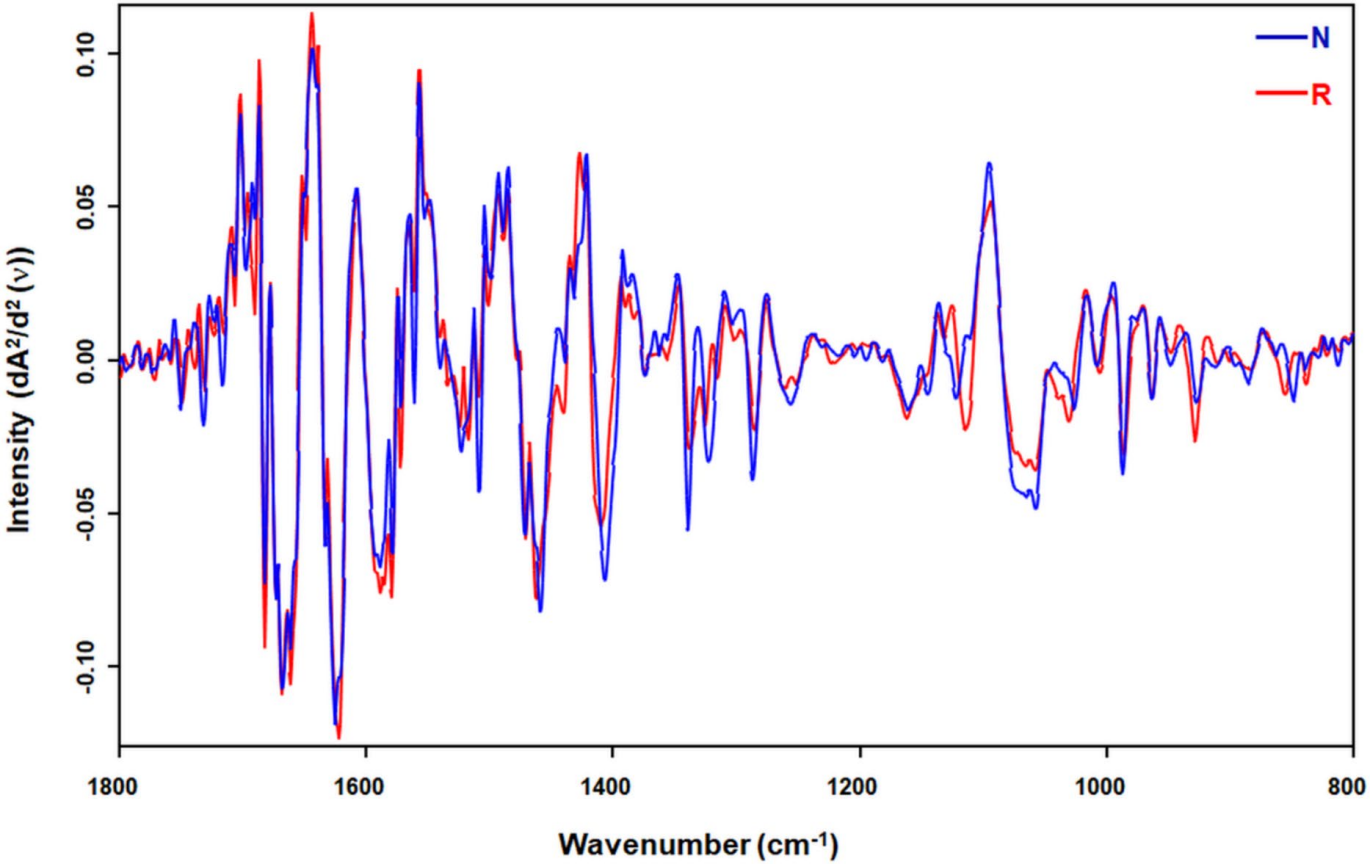
The second-derivative infrared spectra of DNA samples within the spectral range of 1800–800 cm^−1^, derived from the liver tissues of *Nannospalax xanthodon* (N) and *Rattus rattus* (R)

The second-derivative fingerprint spectra of DNA, which span the spectral range of 1800–800 cm⁻^1^, are presented in Fig. 3. This spectral region is particularly significant as it encompasses key vibrational modes associated with the molecular structure of DNA. By applying second-derivative processing, subtle features within the spectra were accentuated, thereby facilitating more precise identification and quantification of the relevant spectral bands.

Furthermore, the calculated spectral bands, along with their corresponding assignments and interpretations, are detailed in Table 1. This table provides a systematic breakdown of the observed bands, enabling a clearer understanding of their biochemical significance. Together, the visual representation in Fig. 3 and the descriptive data in Table 1 offer a comprehensive framework for interpreting the structural attributes of DNA as reflected in its spectral signature. These findings not only enhance our understanding of the molecular composition of DNA but also lay the groundwork for further investigations into its functional dynamics.

**Table 1:** Spectral Band Assignments and Characteristic Parameters of Genomic DNA from *Nannospalax xanthodon* (N) and *Rattus rattus* (R)

Position (cm^−1^)	Assignment	Description
1248–1241	A-DNA	Antisymmetric PO_2_ stretching
963	A-DNA	C–C stretching of the backbone
1222	B-DNA	Antisymmetric PO_2_ stretching
987	B-DNA	Sugar-phosphate backbone C–C stretching
1121	Z-DNA	Z-form DNA
928–927	Z-DNA	Sugar/sugar-phosphate vibrations
1065	Furanose ring	C-O stretching of the backbone, Enhanced in Z-DNA
1058		
1411–1406	N-type sugar	C3-endo sugar puckering, sugar/sugar-phosphate vibrations
886–885		
839–838	S-type sugar	C2-endo sugar puckering, furanose-phosphodiester chain vibrations
1524	Cytosine	In-plane ring vibrations within the cytosine base
1517		
1508		
1286–1285	Cytosine	C5 = C6 ring stretching vibration
1339–1337	Adenosine, thymidine	In anti-conformation, N-type sugar, C3-endo sugar puckering
1325–1322	Guanosine	In syn-conformation, N-type sugar, C3-endo sugar puckering

The spectral bands were assigned according to the literature (Tajmir-Riahi and Theophanides 1985; Banyay and Gräslund 2002; Banyay et al. 2003; Whelan et al. 2011, 2014; Muntean et al. 2013; Zhang et al. 2016; Talari et al. 2017; Gurbanov et al. 2018, 2019)

**Fig. 4:**
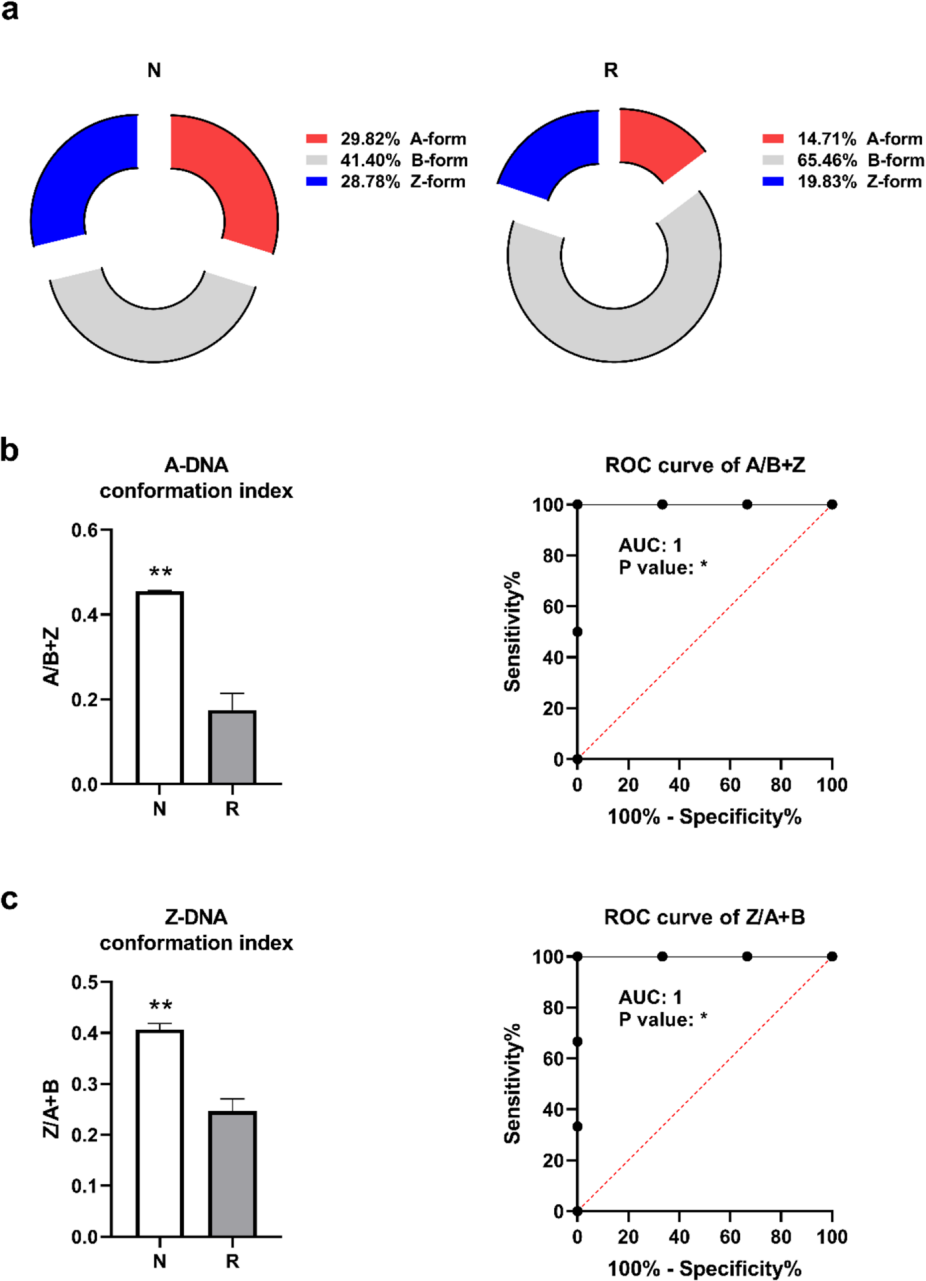
Spectral indices associated with conformational forms in *Nannospalax xanthodon* (N) and *Rattus rattus* (R) DNA samples. **a** Pie chart demonstrating the percentage distribution of different DNA forms. **b** A-DNA (A/B + Z) and **c** Z-DNA (Z/A + B) indices with corresponding ROC curves

A comparative analysis of DNA conformational distributions between *N. xanthodon* and *R. rattus* specimens reveals pronounced interspecific divergence in the prevalence of A-form, B-form, and Z-form configurations (Fig. 4a). In *N. xanthodon*, the A-form conformation constitutes 29.82% of the total DNA structural profile, whereas, in *R. rattus*, this conformation is markedly reduced to 14.71%, representing a significant twofold disparity. The B-form conformation, while dominant in both species, exhibits a striking contrast in its proportional representation. Specifically, *N. xanthodon* displays a B-form prevalence of 41.40%, contrasting sharply with the substantially higher proportion observed in *R. rattus* (65.46%). This differential suggests a pronounced species-specific preference for the B-form conformation, with *R. rattus* exhibiting a 1.6-fold increase relative to *N. xanthodon*. Furthermore, Z-form conformations comprise 28.78% of the structural profile in *N. xanthodon*, compared to 19.83% in *R. rattus*, indicating a relative elevation of non-canonical DNA structures in the former species. Collectively, these data underscore a structural divergence between the two species, characterized by a significant reduction in B-form DNA coupled with elevated A- and Z-form conformations in *N. xanthodon*. The observed disparity in conformational equilibria, particularly the predominance of B-form DNA in *R. rattus*, implies potential species-specific adaptations in DNA topology. Further investigation is warranted to elucidate the mechanistic and evolutionary drivers underlying these conformational preferences. The indices for A- and Z-form DNA conformations were computed by integrating the spectral band intensities associated with total A, total B, and total Z-form conformations, as detailed in Table 1. Notably, the A/B + Z conformation index exhibited a significantly higher value in *N. xanthodon* samples compared to *R. rattus* samples (Fig. 4b). The ROC curve analysis for the A/B + Z index revealed perfect discrimination between *N. xanthodon* and *R. rattus* samples, achieving AUC of 1.0 with a p-value less than 0.05, indicating statistical significance. Similarly, the Z/A + B conformation index was also markedly elevated in *N. xanthodon* samples relative to *R. rattus* samples. Consistent with the findings for the A/B + Z index, the ROC curve for the Z/A + B index demonstrated flawless discrimination, yielding an equally significant AUC value of 1.0 (Fig. 4c). These results underscore the robustness of these indices in differentiating between the two species based on their respective DNA conformational profiles.

**Fig. 5:**
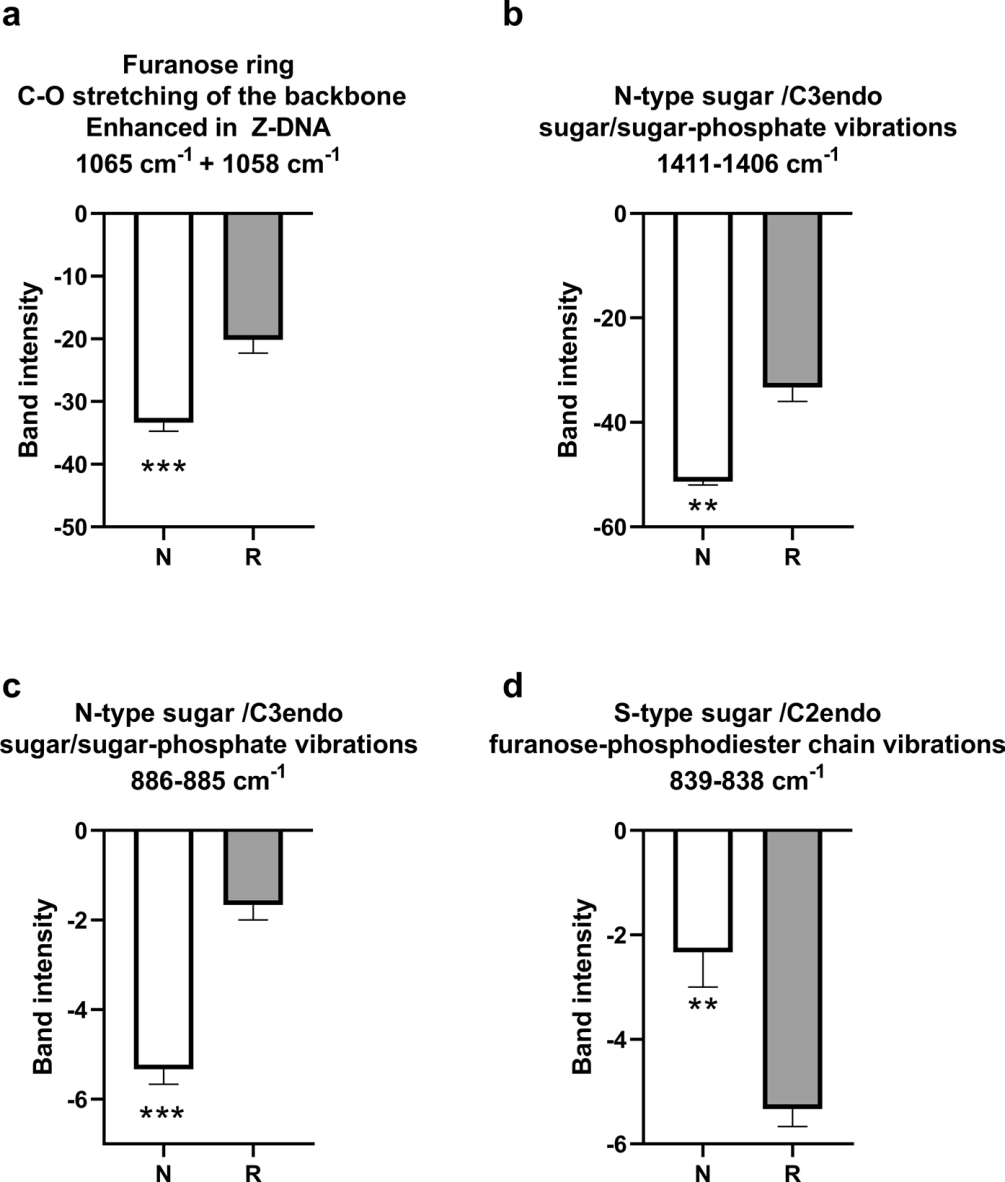
Spectral indices associated with sugar conformational forms in *Nannospalax xanthodon* (N) and *Rattus rattus* (R) DNA samples. The spectral bands demonstrating **a** Furanose ring, **b–c** N-type sugars with C3-endo, and **d** S-type sugar with C2-endo conformations

The comparative analysis of vibrational spectroscopic profiles between *N. xanthodon* and *R. rattus* DNA specimens, as illustrated in Fig. 5, reveals distinct molecular signatures associated with sugar-phosphate vibrational modes. Spectral bands observed at 1065 cm⁻^1^ and 1058 cm⁻^1^ correspond to C-O stretching vibrations of the furanose ring within the DNA backbone. These vibrational modes are well-documented in literature as diagnostic markers of Z-form DNA conformation (Fig. 5a). Quantitative evaluation of band intensities demonstrated a statistically significant elevation (*p < 0.001, denoted by ***) in *N. xanthodon* relative to *R. rattus*. This marked disparity in spectral intensity implies enhanced furanose ring C-O vibrational activity in *N. xanthodon* DNA, suggesting a greater propensity for Z-form structural conformation compared to the *R. rattus* samples.

The pronounced differences in sugar puckering conformations between *N. xanthodon* and *R. rattus* DNA are reflected in Figs. 5b–d. Distinct bands within the 1411–1406 cm⁻^1^ and 886–885 cm⁻^1^ regions correspond to C3-endo puckering modes of N-type sugar rings, characteristic of non-canonical DNA helices other than B-conformation described by Watson and Crick. Quantitative analysis revealed significantly elevated band intensities for these N-type sugar vibrations in *N. xanthodon* compared to *R. rattus*, indicative of enhanced conformational rigidity or population of N-type sugar geometries in the former species. Conversely, the S-type sugar’s C2-endo puckering mode, manifested as a band at 839–838 cm⁻^1^, exhibited a reciprocal trend, with *R. rattus* displaying statistically higher intensity relative to *N. xanthodon*.

**Fig. 6:**
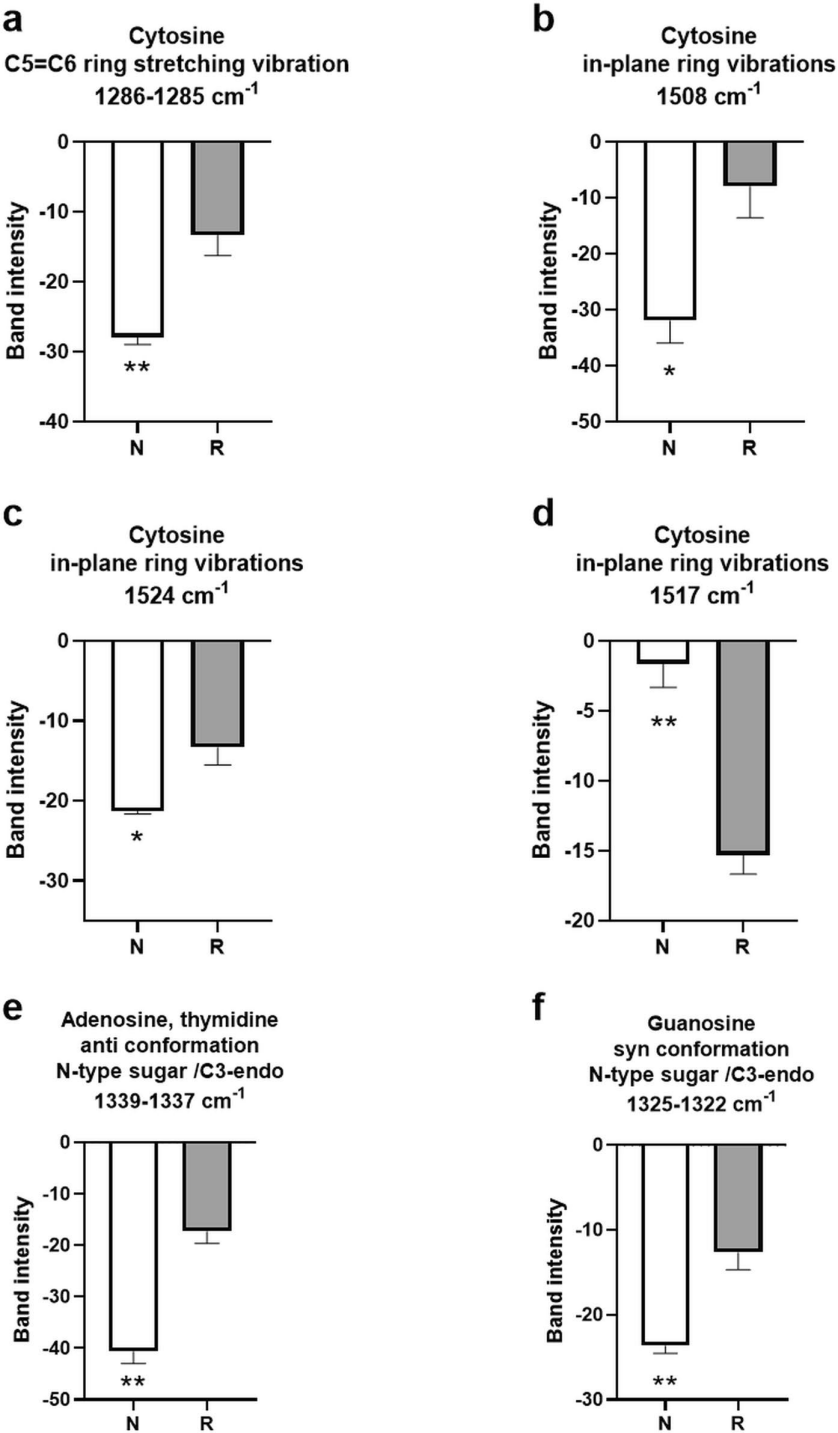
Spectral indices associated with nucleobases conformational forms in *Nannospalax xanthodon* (N) and *Rattus rattus* (R) DNA samples. The spectral bands demonstrating **a–d** cytosine, **e** adenosine, thymidine, and **f** guanosine conformations

The spectroscopic analysis of vibrational modes in nitrogenous purine and pyrimidine bases, as detailed in Fig. 6, reveals marked interspecific divergence in DNA structural dynamics between *N. xanthodon* and *R. rattus*. Quantitative analysis demonstrates a statistically significant elevation in band intensities for *N. xanthodon* DNA at 1286–1285 cm⁻^1^, corresponding to C5 = C6 stretching vibrations of cytosine rings, and at 1508 cm⁻^1^ and 1524 cm⁻^1^, associated with in-plane cytosine ring vibrational modes (Fig. 6a–c). Conversely, *R. rattus* DNA exhibited a pronounced intensity enhancement at 1517 cm⁻^1^, a spectral region similarly linked to cytosine in-plane ring vibrations (Fig. 6d). Spectral bands at 1339–1337 cm⁻^1^, diagnostic of anti-conformations in adenosine and thymidine residues, and at 1325–1322 cm⁻^1^, corresponding to syn-conformational geometries of guanosine, exhibit statistically significant intensity enhancements in *N. xanthodon* relative to *R. rattus* (Fig. 6e–f). These vibrational modes are mechanistically linked to C3-endo puckering of N-type sugar moieties, a structural feature associated with non-canonical helical conformations.

## Discussion

DNA conformation has become a focal point of research following the discovery of DNA polymorphism. In addition to B-DNA, recognized as the canonical DNA form, alternative structural conformations, including hairpins, triplexes, cruciforms, left-handed Z-DNA, G-quadruplexes, and A-motifs have been identified (Whelan et al. 2014). Under physiological conditions, the predominant B-DNA adopts a right-handed antiparallel double-helical structure, with a full 360° helical turn encompassing 10 nucleotide pairs. Certain nucleotides within this structure are oriented perpendicular to the helix axis (Baker and Bowers 2007). Among alternative DNA forms, Z-DNA has garnered significant scientific interest due to its unconventional structure and potential roles in biological processes (Pohl and Jovin 1972; Bhanjadeo et al. 2022). Z-DNA, a left-handed helix stabilized in regions with alternating purine-pyrimidine sequences, features guanine residues in the syn-conformation across both base pairs, contrasting with the canonical anti-conformation observed in B-DNA (Subramani et al. 2019). Unlike B-DNA, where bases uniformly adopt the anti-conformation, Z-DNA exhibits an alternating syn-anti pattern (Nial et al. 2024a). This conformation, coupled with the zigzag arrangement of its sugar-phosphate backbone, results in a greater inter-base-pair distance compared to B-DNA (De Rosa et al. 2010). Z-DNA is implicated in critical cellular processes such as transcriptional regulation and double-strand break repair, thereby influencing various human diseases (Nial et al. 2024b).

In vitro studies demonstrate that cytosine methylation can induce structural alterations in DNA, reducing its free energy and promoting B-to-Z-DNA transitions (Plass and Soloway 2002). However, the molecular mechanisms underlying methylation-driven conformational dynamics between B- and Z-DNA remain poorly characterized, despite established correlations between DNA methylation and structural changes (Temiz et al. 2012). Methyl-sugar interactions represent another determinant of conformational shifts in the DNA backbone. These interactions modulate DNA structure and flexibility, inducing intrinsic helical bending (Dršata and Lankaš 2013). Furthermore, methyl-sugar interactions adversely affect DNA torsional and bending rigidity, as well as structural flexibility. Although DNA methylation is known to influence overall DNA architecture, its precise effects on backbone conformation remain unresolved (Liebl and Zacharias 2019). DNA topological diversity also arises from variations in sugar and backbone conformations, glycosidic bond orientation, steric effects, and differential base-pairing flexibility. Z-DNA, a structure favored by GC-rich alternating sequences (Nayak et al. 2016; Bhanjadeo and Subudhi 2019), diverges from A- and B-DNA: purine residues (G) adopt a syn orientation with C3-endo sugar pucker, while pyrimidine residues (C) assume an anti-conformation with C2-endo puckering (Banyay et al. 2003).

Non-B DNA-forming sequences in genomes contribute to genomic instability by interfering with DNA replication and transcription (Bacolla and Wells 2004; Bhanjadeo et al. 2017). Previous studies have reported that alternative DNA conformational forms induce dysregulation of cancer-associated genes in malignancies such as myeloma, leukemia, and lymphoma (Bacolla and Wells 2009; Wells 2013; Bhanjadeo et al. 2019). Abnormal DNA methylation, a universal hallmark of cancer, disrupts gene expression and plays a critical role in cancer development and progression (Mahmoud and Ali 2019). Cytogenetic analyses of various cancers, particularly hematological malignancies, reveal that a significant proportion of patients exhibit nonrandom chromosomal breakage and translocation events (Lieber et al. 2006). DNA breaks occurring during conformational processes may culminate in chromosomal translocations, gene duplications, inversions, and deletions, hallmarks of cancer cells (Nambiar et al. 2008). Although the precise origins or initiating triggers of abnormal methylation remain unknown, this phenomenon is hypothesized to emerge as an age-related process (Issa 2014).

Blind mole rats are recognized as exceptional models for aging and cancer research due to their extended lifespan, absence of age-associated phenotypic deterioration, and resistance to spontaneous tumorigenesis (Lagunas-Rangel 2018). Members of the Spalacidae family (which has two genera, *Nannospalax* and *Spalax*) inhabit subterranean environments characterized by hypoxic and hypercapnic conditions, which induce toxic stress and cellular damage in surface-dwelling rodents (Manov et al. 2020; Bugarski-Stanojević et al. 2024).

Although species belonging to the genus *Spalax* are frequently studied, research on cancer resistance and aging in species of the genus *Nannospalax* is relatively scarce. Remarkably, *Spalax* survives ~ 3% O_2_ under laboratory conditions for up to 14 h, whereas rat survives for only ~ 2–3 h. Furthermore, *Spalax* exhibits a lifespan exceeding 20 years, far surpassing the 4–5-year lifespan of rats (Malik et al. 2016). Comparative studies between *Spalax ehrenbergi* and *Rattus norvegicus* species have identified divergent expression patterns in hypoxia-responsive genes, including hemoglobin, myoglobin, neuroglobin, cytoglobin, erythropoietin receptors, vascular endothelial growth factor, and hypoxia-inducible factor 1α (Shams et al. 2004; Avivi et al. 2005). Studies on *N. xanthodon* have investigated the role of Janus kinase-signal transducer and activator of transcription (JAK-STAT) signaling pathways and cytokine-mediated signaling pathways in cellular aging processes. These inflammatory factors have been highlighted for their potential contributions to the species' high resistance to aging and cancer (Inci et al. 2022). Additionally, 16S rRNA metabarcoding analyses of *N. xanthodon*'s gut microbiota revealed a high abundance of bacterial families associated with enhanced performance and longevity (Solak et al. 2023). Similarly, the *Muribaculaceae* bacterial family, which is associated with longevity, was identified as the most dominant bacterial taxon in fecal samples of *Spalax leucodon* (Sibai et al. 2020).

This study elucidates the conformational differences in DNA between the subterranean-dwelling *N. xanthodon* and the surface-dwelling *R. rattus*. Employing IR spectroscopy, we conducted a quantitative analysis of the structural adaptations in *N. xanthodon* DNA, which has evolved under specific selective pressures associated with the challenging subterranean environment. Our findings revealed that *N. xanthodon* exhibits a higher content of A- and Z-DNA conformations compared to *R. rattus*. Additionally, the sugar-phosphate backbone vibrations demonstrated a greater prevalence of N-type (C3-endo) sugar puckering in *N. xanthodon*, alongside a reduction in S-type (C2-endo) puckering, and vice-versa for *R. rattus*. The results also suggest that B-DNA is more predominant in *R. rattus*, reflecting distinct evolutionary adaptations to their respective ecological niches. These observations align with established structural correlations: N-type sugar puckering (C3-endo) is mechanistically linked to A-DNA topology, while S-type puckering (C2-endo) predominates in B-form duplex DNA (Egli 2018). On the other hand, structural, biochemical, and computational studies show the coexistence of C3-endo and C2-endo sugar puckers in Z-DNA (Peter Slickers 1999; Egli 2018). These conformations are critical for stabilizing the left-handed helix in Z-DNA. Indeed, this structural flexibility allows Z-DNA to act as a "flipon", dynamically switching between conformations to regulate chromatin remodeling and transcription re-initiation (Beknazarov et al. 2024; Sahayasheela et al. 2025).

Environmental stress factors caused by subterranean life play a significant role in the evolution of *N. xanthodon*. These stress factors are identified as darkness, energy constraints, low productivity, nutrient scarcity, soil and rock structure, hypoxia, hypercapnia, and high pathogenicity (Nevo et al. 1994). *Spalax ehrenbergi* has developed numerous adaptation strategies to survive under hypoxic conditions. One such strategy involves mutations in the tumor suppressor gene P53, which supports the transcription of DNA repair genes over apoptosis genes. The transcriptional regulation of this gene is part of the intracellular control mechanism, governing cellular responses to various stress conditions, including DNA damage and hypoxia, leading to growth arrest or apoptosis (Ashur-Fabian et al. 2004). Research conducted over the last decade has revealed that dysregulation of DNA repair pathways may significantly contribute to hypoxia-induced genetic instability (Shams et al. 2013). Under normal conditions, P53 halts cell proliferation and initiates DNA repair upon detection of DNA damage. However, under hypoxic conditions, mutations in P53 increase the transcription of DNA repair genes over apoptotic genes, thereby helping to prevent cell death. The long lifespans and low cancer rates observed in *Spalax ehrenbergi* indicate that this adaptive strategy functions effectively (Nasser et al. 2005). It has been reported that *Spalax carmeli* fibroblasts exhibit successful resistance to genotoxic stress through effective DNA repair compared to *Rattus* fibroblasts. These findings are partially consistent with *Spalax*’s resistance to carcinogenesis (Domankevich et al. 2016, 2018). The identification of DNA conformation in eukaryotic cells holds great importance for understanding how certain cells maintain their functionality in response to environmental stress (Whelan et al. 2011). Therefore, the dominant forms detected in *N. xanthodon* DNA support previous studies on *Spalax* within the context of their DNA structural properties.

By examining vibrational modes associated with nitrogenous purine and pyrimidine bases, this study uncovers species-specific patterns that reflect differences in base-stacking interactions, hydrogen-bonding dynamics, helical torsional stress, and conformational preferences. These findings highlight the nuanced interplay between nucleobase vibrational states and macromolecular conformation. A key observation from the data is the marked difference in cytosine-related vibrational bands between the two species. In *N. xanthodon*, there is a statistically significant elevation in band intensities at 1286–1285 cm⁻^1^ (C5 = C6 stretching vibrations) and 1508 cm⁻^1^ and 1524 cm⁻^1^ (in-plane cytosine ring modes). Conversely, *R. rattus* exhibits an intensity enhancement at 1517 cm⁻^1^, accompanied by attenuation of the 1524 cm⁻^1^ band. This reciprocal band intensity relationship, characterized by concomitant attenuation of the 1524 cm⁻^1^ band and spectral downshifting to 1517 cm⁻^1^ in *R. rattus,* suggests a species-specific redistribution of vibrational energy within cytosine residues (Banyay and Gräslund 2002). The observed spectral shifts could suggest changes in electronic environments or structural strain within nucleobase arrangements, possibly reflecting variations in base-stacking interactions, hydrogen-bonding patterns, or helical stress. The differences in vibrational signatures might be associated with altered stabilization of DNA secondary structures or differing epigenetic modification patterns, indicating potential connections between nucleobase vibrational behavior and larger-scale molecular conformations across these phylogenetically distinct species.

Further analysis of adenosine, thymidine, and guanosine vibrational modes reveals additional interspecific differences. Bands at 1339–1337 cm⁻^1^ (anti-conformations of adenosine and thymidine) and 1325–1322 cm⁻^1^ (syn-conformations of guanosine) show significantly higher intensities in *N. xanthodon* compared to *R. rattus*. The marked elevation in band intensities for *N. xanthodon* suggests a preferential stabilization of anti/syn glycosidic torsion angles and N-type sugar pucker geometries, potentially reflecting enhanced populations of A- and/or Z-form DNA topologies. The interspecific differences in conformational tendencies could be influenced by species-specific variations in base-stacking interactions, which may alter torsional strain or potential differences in chromatin compaction dynamics. The spectroscopic trends appear consistent with our evidence of increased Z-form DNA propensity in *N. xanthodon*. Conversely, the reduced signal intensities in *R. rattus* suggest a higher proportion of B-DNA conformations, structures often linked to transcriptionally active chromatin states and typical genome organization patterns.

The previous study revealed that the base significantly affects the conformational energetics of nucleosides, with deoxycytidine showing particularly distinct properties compared to other nucleosides. Deoxycytidine's intrinsic conformational energetics favor the A-form of DNA over the B-form by 2.3 kcal/mol, and it also shows a greater propensity to accommodate Z-DNA conformations compared to deoxythymidine. The research indicates that cytosine's unique properties at the nucleoside level contribute to GC-rich sequences favoring both A-DNA and Z-DNA forms more than AT-rich sequences. Additionally, the study finds that energy barriers between different sugar-puckering conformations vary depending on the base, with pyrimidines generally showing lower barriers than purines in the context of B-DNA. These findings suggest that base composition, in addition to sequence, plays a crucial role in determining DNA conformation and dynamics (Foloppe and MacKerell 1999). The study by Temiz et al. (2012) investigated the role of cytosine methylation on the intrinsic dynamics of B- and Z-DNA through molecular dynamics simulations. The main findings revealed that methylation significantly destabilized the BII state relative to the BI state in B-DNA, particularly through Gp5mC steps, and decreased the free energy difference between B- and Z-DNA, suggesting a lower energetic barrier for the B-to-Z transition in methylated DNA (Temiz et al. 2012).

A recent study presented universal epigenetic clocks applicable across mammalian species and tissues, developed using DNA methylation profiles from 11,754 samples spanning 185 species. These clocks estimate chronological and relative age with high accuracy (r > 0.96) and reveal conserved aging-related cytosine methylation changes, particularly at polycomb repressive complex 2-binding sites near genes linked to development, cancer, and longevity. The clocks demonstrated their biological relevance by correlating age deviations with human mortality risk, somatotropic axis mutations in mice, and caloric restriction effects. Additionally, they provide insights into epigenetic age reversal via reprogramming and link aging to conserved developmental processes (Lu et al. 2023). Another study demonstrated that DNA methylation-based epigenetic clocks accurately estimate chronological age in wild roe deer (*Capreolus capreolus*), best describing the relationship when including juveniles, reflecting accelerated epigenetic changes during growth phases (Lemaître et al. 2022), consistent with patterns observed in humans (Horvath 2013). Sex-specific analyses revealed that older females exhibit slower biological aging than males, linked to differential methylation at loci such as *POU3F3*, implicated in neuronal development. Environmental and life-history factors also influenced aging trajectories: juvenile body mass positively correlated with epigenetic acceleration, suggesting growth-longevity trade-offs (Metcalfe and Monaghan 2003; Lemaître et al. 2022).

Three new hallmarks, disabled macroautophagy, chronic inflammation, and dysbiosis, were introduced to better capture the complexity of aging processes. Disabled macroautophagy was highlighted as a critical mechanism affecting organelle turnover and contributing to age-related decline, with evidence showing that autophagy-related gene expression decreases with age in humans and rodents (López-Otín et al. 2023). Chronic inflammation and dysbiosis were also identified as significant factors influencing aging, with gut microbiota alterations linked to various age-related diseases. The interdependence of these hallmarks suggests that targeting any single hallmark can influence others, emphasizing the need for comprehensive approaches in developing anti-aging interventions (López-Otín et al. 2023). *Spalax* have an extraordinarily long lifespan (~ 20 years in captivity) without clear age-related changes and show no recorded cases of spontaneous tumor development. Living in underground burrows, they face multiple stressors like hypoxia, hypercapnia, and high pathogen exposure, yet have evolved several adaptive mechanisms such as low metabolic rates, enhanced DNA repair capabilities, unique p53 gene mutations, and over-expression of tumor-suppressor genes like A2M. These adaptations help them resist cellular damage and cancer, providing valuable insights into potential human cancer prevention and therapeutic strategies (Lagunas-Rangel 2018). *Spalax* fibroblasts exhibited significant telomere shortening with cell passages, similar to rats, but maintain longer average telomere lengths and have lower telomerase activity (Azpurua and Seluanov 2013). Notably, senescent *Spalax* cells display significantly less DNA damage, particularly at telomeres, compared to rat cells. The expression of shelterin complex components, which protect telomeres, generally declined with cell passages in both species, but more pronounced decreases were observed for shelterin member proteins, TIN2 (TRF1-interacting nuclear protein 2) and TPP1 (telomere-binding protein POT1-interacting protein 1) in *Spalax*. These results suggest that *Spalax* has evolved unique genome protection strategies, including efficient DNA repair mechanisms and maintenance of telomere integrity without relying on telomere elongation, which may contribute to its exceptional longevity and healthy aging. This indicates that telomere integrity maintenance, rather than length maintenance, is a crucial factor in *Spalax*'s aging process and cancer resistance (Adwan Shekhidem et al. 2023). The study by Manov et al. (2020) revealed that *Spalax*, exhibits cellular senescence without a senescence-associated secretory phenotype (SASP), unlike human and mouse fibroblasts. Despite showing typical senescence markers like proliferative arrest and increased p21 and p53 expression, *Spalax* cells do not secrete pro-inflammatory factors such as IL6, IL8, and others. This absence of SASP is linked to efficient DNA repair mechanisms that prevent persistent DNA damage and suppress inflammation. Additionally, the IL1 pathway, a key regulator of SASP, appears impaired in *Spalax*, further reducing inflammatory responses. These findings suggest that *Spalax* has evolved to decouple inflammation from senescence, providing insights into aging and cancer resistance (Manov et al. 2020).

The longevity and cancer resistance observed in species like *N. xanthodon* and *H. glaber* are influenced by a combination of genetic, molecular, and environmental factors. Key mechanisms include enhanced DNA repair pathways, such as base-excision repair and nonhomologous end-joining, which correlate positively with lifespan across various organs. Metabolic regulation plays a crucial role, with long-lived species often displaying lower metabolic rates, reduced oxidative stress, and altered lipid metabolism, including higher levels of sphingomyelins and specific triacylglycerols while having lower levels of polyunsaturated fatty acids. Additionally, genes involved in cell cycle regulation and apoptosis, such as TP53 and its related pathways, show distinct expression patterns favoring cell cycle arrest over apoptosis, particularly under hypoxic conditions. The interplay between these genetic and metabolic factors, alongside efficient genome maintenance systems, contributes significantly to the prevention of cellular damage and aging, thereby supporting healthy longevity and resistance to diseases like cancer (Ma and Gladyshev 2017). The previous study demonstrated that the blind mole rat (*Spalax*) exhibits remarkable resistance to both spontaneous and chemically-induced cancers, as evidenced by experiments where *Spalax* showed minimal tumorigenesis when exposed to potent chemical carcinogens, unlike mice and rats that developed tumors. Notably, normal fibroblasts from *Spalax* were found to inhibit growth and induce death in various human cancer cell lines through direct interaction or soluble factors, without affecting non-cancerous cells. This anti-cancer effect was also observed in another subterranean species, *H. glaber*, but not in above-ground rodents like mice, rats, or *Acomys.* The authors suggest that *Spalax*'s cancer resistance may be linked to its adaptation to hypoxic environments, potentially involving unique antioxidant mechanisms and tumor suppressor activities (Manov et al. 2013). Malik et al. (2016) conducted a cross-species brain transcriptome analysis to investigate the mechanisms underlying the remarkable hypoxia tolerance, cancer resistance, and longevity of the blind mole rat (*Spalax*). Compared to *Rattus norvegicus*, *Spalax* exhibited significantly higher baseline mRNA levels of genes involved in DNA damage repair and genome maintenance, including those related to homologous recombination, base excision repair, and the Fanconi anemia pathway. Additionally, *Spalax* showed lower expression of genes associated with oxidative phosphorylation and mitochondrial metabolism, suggesting a reduced reliance on aerobic respiration, which may help mitigate oxidative stress. The study proposes that these transcriptional adaptations protect *Spalax* from replication stress and DNA damage accumulation during hypoxia-reoxygenation cycles, contributing to its enhanced genome stability, cancer resistance, and extended lifespan (Malik et al. 2016). Notably, long-lived rodents have independently evolved enhanced tumor suppressor mechanisms, such as repressed telomerase activity in large-bodied species and alternative telomere-independent controls in small, long-lived species, which correlate with slow cell proliferation rates and increased cancer resistance (Gorbunova et al. 2014).

## Conclusion

This study provides novel insights into the biological processes underlying the correlation between DNA structural polymorphism and the exceptional longevity phenotype exhibited in *N. xanthodon*. Comparative genomic analyses reveal pronounced divergence in DNA conformational profiles between long-lived *N. xanthodon* and shorter-lived *R. rattus*, implicating topological variation as a potential modulator of lifespan extension. A critical discovery is the significantly elevated prevalence of non-canonical A-DNA (29.82%) and Z-DNA (28.78%) conformations in *N. xanthodon* relative to *R. rattus* (14.71% A-form, 19.83% Z-form), despite B-DNA predominance in both species. Concomitant alterations in sugar pucker dynamics further corroborate this divergence: *N. xanthodon* exhibits increased N-type (C3-endo) puckering, a configuration thermodynamically compatible with A- and Z-DNA topologies, alongside reduced S-type (C2-endo) puckering characteristic of B-DNA. Spectroscopic analyses additionally identify species-specific nucleobase vibrational modes, with *N. xanthodon* demonstrating enhanced cytosine ring vibrations and perturbed base-stacking interactions.

The substantial Z-DNA enrichment in *N. xanthodon* holds particular significance, given this left-handed conformation’s established role in transcriptional control and double-strand break repair. The distinctive structural polymorphism observed in *N. xanthodon,* marked by helical diversity, sugar pucker shifts, and nucleobase dynamics proposes DNA topology as a potential biomolecular determinant of longevity. While these findings substantiate a putative association between conformational plasticity and lifespan extension, causal relationships remain to be established. Future research must delineate how these structural features synergize with epigenetic, proteostatic, and metabolic networks to mediate healthy aging.

These findings contribute to our understanding of how vibrational spectroscopy can serve as a powerful tool for probing DNA structural dynamics at the molecular level. The ability to detect subtle differences in nucleobase vibrational modes offers new opportunities to explore the relationship between DNA conformation and biological function. Future research could expand on these results by investigating the impact of environmental factors, such as temperature or oxidative stress, on vibrational signatures. Additionally, integrating computational modeling with experimental data could provide deeper insights into the energetic landscapes governing DNA conformational transitions.

### Limitations of the study

Although our findings provide valuable insights into genomic divergence between *N. xanthodon* and *R. rattu*s, we emphasize that the limited sample size (three individuals per species) constrains the scope of our conclusions. This sample size is insufficient to characterize intraspecific genetic diversity, population structure, or demographic history within either species. However, our analysis intentionally focuses on cross-species comparisons and avoids extrapolating results to within-species processes, as such inferences would require broader sampling across geographic ranges and ecological contexts. Future work prioritizing intraspecific resolution should incorporate larger cohorts, balanced with ethical and logistical considerations for specimen acquisition, to disentangle population-level dynamics in these systems.

While our study identifies unique DNA conformational features in *N. xanthodon* and contextualizes them within the documented longevity of species, we acknowledge that direct causal evidence linking these structural variations to longevity remains beyond the scope of the current work. The observed conformational landscape suggests a potential contribution to longevity, consistent with established genomic stability and repair paradigms in long-lived species. Further functional studies (e.g., comparative epigenomic profiling or targeted mutagenesis) are needed to validate this hypothesis. Moreover, future studies employing advanced biophysical techniques, such as proton Nuclear Magnetic Resonance (NMR) and circular dichroism (CD) spectroscopy, could provide additional validation of the predicted abundance of A- and Z-DNA conformations in *N. xanthodon*. Furthermore, integrating dye-binding assays could provide complementary evidence for sequence-specific or stress-induced Z-DNA formation, further validating conformational predictions and enhancing the resolution of dynamic structural transitions. Finally, functional studies are needed to confirm immune adaptations and their evolutionary significance in *N. xanthodon*. All these methods would offer direct evidence of the structural transitions and complement the findings reported here.

## Data Availability

No datasets were generated or analysed during the current study.
